# Fatal Extraction of Tooth

**Published:** 1914-06

**Authors:** 


**Fatal Extraction of Tooth.** The Dublin Journal of Medical Science, April 1914, cites the case of a young man. Nitrous oxid was administered, but was not regarded as the cause of death. Artificial respiration was used in vain. Post mortem examination showed tuberculosis of upper cervical vertebrae and base of skull and fracture of the 4th cervical vertebra. The resulting spinal compression was considered as the cause of death, fractures being predisposed to by the tuberculous disease.

